# Rainwater-Removal Image Conversion Learning with Training Pair Augmentation

**DOI:** 10.3390/e25010118

**Published:** 2023-01-06

**Authors:** Yu-Keun Han, Sung-Woon Jung, Hyuk-Ju Kwon, Sung-Hak Lee

**Affiliations:** School of Electronic and Electrical Engineering, Kyungpook National University, 80 Deahakro, Buk-Gu, Daegu 702-701, Republic of Korea

**Keywords:** GAN, Pix2pix, augmentation learning, rainwater removal, image-to-image learning

## Abstract

In this study, we proposed an image conversion method that efficiently removes raindrops on a camera lens from an image using a deep learning technique. The proposed method effectively presents a raindrop-removed image using the Pix2pix generative adversarial network (GAN) model, which can understand the characteristics of two images in terms of newly formed images of different domains. The learning method based on the captured image has the disadvantage that a large amount of data is required for learning and that unnecessary noise is generated owing to the nature of the learning model. In particular, obtaining sufficient original and raindrops images is the most important aspect of learning. Therefore, we proposed a method that efficiently obtains learning data by generating virtual water-drop image data and effectively identifying it using a convolutional neural network (CNN).

## 1. Introduction

As interest in artificial-intelligence-based object recognition has increased, autonomous vehicles using this technology are actively being developed. One of the core technologies used in autonomous vehicles is securing a good driving view from the camera sensor rather than from the driver’s vision. However, owing to climate change caused by bad weather, securing the visibility of the driving route is often difficult. In particular, during rainfall, raindrops form on the lens, and an autonomous driving accident may occur because of the failure to recognize obstacles in the front. Since this can lead to casualties, it can be said that the technology to remove foreign substances from the camera lens is important for autonomous driving technology.

For image learning for water-drop removal, a dataset of pairs of images with no foreign objects on the lens and images with raindrops on the same field of view is required. Using these two images, it is necessary to learn to detect and remove the raindrops. Learning using existing generative adversarial networks (GAN) requires a single-image set rather than an image pair; hence, there is a limitation in raindrops removal because the desired conditions cannot be specified [1,2,3,4,5,6]. On the other hand, the cycleGAN and Pix2pix are sorts of generative models for image-to-image translation to map input images to output images using image datasets. Like a general GAN, there is a generator that forms a new image and a discriminator that separates the generated image from the actual image. However, while a general GAN requires one image input, the cycleGAN and Pix2pix have an image-to-image translation function that requires two images as input.

**Figure 1:**
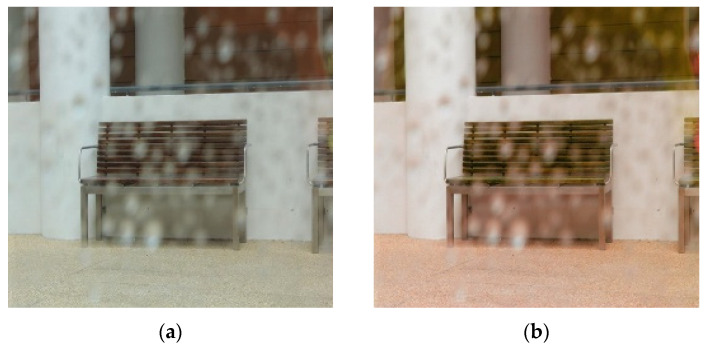
Images of raindrops input and removal result using cycleGAN: (**a**) raindrops image and (**b**) cycleGAN.

Because it does not require pair images for the same scene, cycleGAN has an advantage owing to its easier dataset acquisition compared to Pix2pix [1,7], which uses pair images. However, as shown in Figure 1, the resulting image converted using the cycleGAN learning method has the disadvantage that the color or texture change of the image is sensitively generated and that the required raindrop-removal effect does not appear well. Thus, existing raindrop detection and removal methods detect the droplet area well but do not efficiently remove the raindrops of corresponding areas well and generate a lot of errors compared to the actual target image. A method for detecting and removing raindrops using a stereo image or a specially designed optical shutter has been proposed, but it has the disadvantage that it cannot be applied to a single input image taken with a general camera [8,9,10,11]. In addition, Pix2pix and cycleGAN, which are attracting attention as conditional training methods, can be used to remove raindrops for a single input image. However, in the experimental results, there is a disadvantage that only some parts of raindrops are removed from the image or that unnecessary noise is generated, and when the raindrops are relatively large or dense, the image improvement process is not performed well.

In this paper, we propose a method for improving raindrops removal through a three-step complementary learning based on the Pix2pix model. Pix2pix is a model that learns with paired images and has the characteristic of comprehending differences between corresponding images by comparing images [1]. This indicates that it is more advantageous for the removal of water drops. However, because it is difficult to obtain sufficient pair datasets, in this study, we first train the Pix2pix image generator that creates raindrops based on the previously obtained dataset for data augmentation learning. In the images generated as a result of primary learning, there are images in which raindrops are clearly created, while images that do not also exist. Because the model is trained with an insufficient dataset, the quality of the droplets generated from the trained model is uneven.

Therefore, to classify images with well-formed raindrops, convolution neural network (CNN)-based learning [12] is introduced to effectively classify images with well-formed raindrops and those without raindrops. Finally, the classified images were again input together with the existing dataset as a learning image in Pix2pix to supplement the learning to remove raindrops.

Therefore, the water-drop-removal model using the Pix2pix is trained in the following three steps: the droplet data augmentation learning, classification learning using CNN, and droplet removal learning. This model only learned a simple droplet-removal transformation. Compared with the rough resulting image, it can be seen that the raindrops part of the image is better removed.

## 2. Related Research

### 2.1. Pix2pix Conversion Training

**Figure 2:**
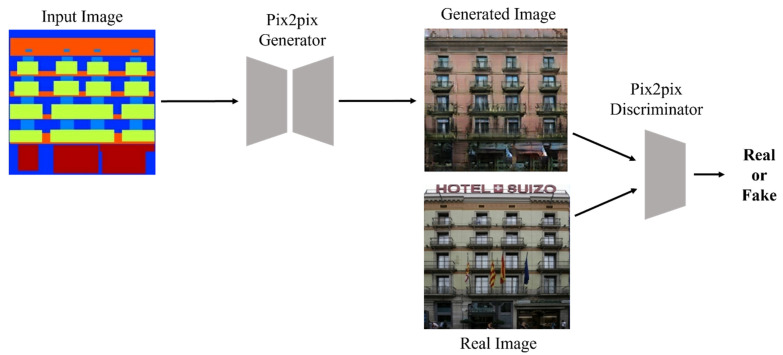
Pix2pix block diagram.

Pix2pix model is an image-to-image translation method that converts an image of one domain into an image of another domain [1]. Pix2pix is a type of generative model that aims at image-to-image conversion, i.e., mapping input images to output images using an image dataset. Just like other GANs, there is generator that forms new images from input images and a discriminator that separates the artificial images generated by the generator from the actual image. Pix2pix is also a type of generative model. Like GAN, the discriminator learns to classify real data as real and artificial data as fake, while the generator learns to discriminate between the data formed by the generator and the real data. In other words, the generator tries to lower the classification performance of the discriminator, and the discriminator has the nature of adversarial learning to form a structure in which each other develops competitively in the direction of increasing the classification performance. As shown in Figure 2, while a general GAN generator requires a noise vector as an input, Pix2pix additionally requires an input image rather than a noise vector [1,6,7].

In addition, the discriminator requires two input images. One is an image generated by the generator, and the other is a target image. By providing these two paired images to the discriminator, the discriminator compares the provided paired images and determines whether the image is generated or real. The loss function of Pix2pix is determined using the following equation:

(1) $$L_{GAN}\left(G,D\right)=E_{y}[\log D\left(y\right)]+E_{x,z}[\log(1-D\left(G\left(z\right)\right)],$$

(2) $${ℒ}_{cGAN}\left(G,D\right)=E_{x,y}[\log D\;\left(x,y\right)]+E_{x,z\;}[\log\left(1-D_{Y}\left(x,G\left(x,z\right)\right)\right)],$$

(3) $${ℒ}_{L1}\left(G\right)=E_{x,y}\|y-G{\left(x\right)\|}_{1},$$

(4) $$G=argmin_{G}max_{D}L_{cGAN}\left(G,D\right)+\lambda L_{L1\;}\left(G\right),$$

where *G* and *D* denote the generator and discriminator, respectively, and *G*(*x*,*z*) is the image generated by the generator. The factor E represents the expected value. x is the training sample data, and y is the expected value generated by the generator receiving the sample data x as an input. Both for the GAN and cGAN, the latent vector *z* forms new data through the generator, and the discriminator compares it with the real image to determine if it is real or fake. The principles of learning in the same direction and the discriminator learning antagonistically in the direction of maximizing the probability of discrimination in order to make a correct discrimination are the same. However, as mentioned earlier, cGAN additionally provides sample data *x* as an input to the generator. In the existing GAN, learning was performed assuming that the latent *z* was just a latent vector sampled from a gaussian distribution, but as in cGAN, if sample data *x* is provided in addition to the latent vector, the random noise vector *z*, that is, the noise *z* affecting the generated image can occur while being ignored, a random noise vector *z* is provided by setting dropout in the learning and testing processes to provide some stochastic properties. Pix2pix comprises an adversarial learning neural network. In other words, the generator is trained to prevent the discriminator from properly discriminating the image generated when the input image is inserted, and the discriminator is trained to properly discriminate the images of the two regions. Thus, if only the adversarial loss is used, as in Equation (2), the image looks slightly blurry; therefore, L1 loss is used, as in Equation (3) [13,14]. L1 loss is used to calculate the similarity between the ground-truth output and the generated output. In other words, this is minimized to generate a general image by obtaining the pixel distance between the target and the generated images. Therefore, the final loss function of Pix2pix is calculated using Equation (4) by combining Equations (2) and (3). Additionally, the weight of each loss can be adjusted by setting a lambda (λ).

### 2.2. CNN Classification Training

The CNN learning model is a useful algorithm for finding patterns to analyze images [12]. A CNN consists of a convolutional layer that extracts features and a fully connected layer that classifies images. The convolutional layer, which is a feature extraction area, consists of a filter that extracts the features for each pixel and an activation function that converts the filtered value into a nonlinear value. Convolutional networks are driving advances in recognition. Convolutional networks are not only improving for whole-image classification but also making progress on local tasks with structured output [15,16,17,18].

**Figure 3:**
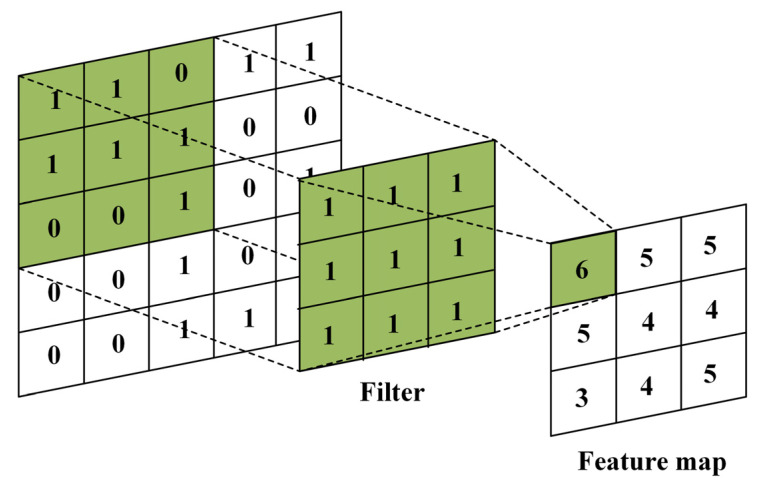
Application method of the convolutional layer filter works.

The data input to the CNN is composed of a three-dimensional tensor of height, width and channel, which is displayed in the form of a matrix. One convolutional layer consists of as many filters as the number of channels in the image. The output of the convolutional layer is determined by applying a filter to each channel. In general, a filter in the form of a square matrix, such as a 3 × 3 or 5 × 5 matrix, is used. When a filter is applied to each pixel, as shown in Figure 3, a feature map can be obtained from the calculation. The feature is the result of filter being traversed at a specified interval, and each pixel value is calculated and outputted using the sum of element-wise multiplication. In this case, the filter interval can be changed by designating a stride. Figure 3 shows the calculated feature map with a stride set to one, and the filter moves by one to execute the pixel operation. Fixed-size convolutional networks have been applied to many applications, among others, handwriting recognition [16,17], machine-printed character recognition [18], online handwriting recognition [19] and face recognition [20].

However, as mentioned earlier, the size of the image outputted through the convolution layer gradually decreases, which means that the information about the pixels disappears. This problem can be solved by using padding. Padding means that the size of the input and output images can be similarly adjusted by adding a pixel with a specific value to the edge of the image. In other words, it is possible to prevent the size of the output image of the convolutional layer from being reduced by adding a specific value.

In addition, CNN has a pooling layer. The pooling layer receives the output value of the convolution layer as input and used it to reduce the size of the data or emphasize specific data. The pooling layer is mainly composed of max pooling and average pooling. In maximum pooling, the maximum value in the data is collected and expressed, whereas average pooling is a method used for expressing the values by averaging them in the data area. Equations (5) and (6) express the output image sizes of the convolutional layers.

(5) $$O_{height}=\frac{\left(I_{height}+2P-KH\right)}{S}+1,$$

(6) $$O_{width}=\frac{\left(I_{width}+2P-KW\right)}{S}+1,$$

where *O_height_* and *O_width_* are the height and width of the output data, respectively, and *I_height_* and *I_width_* are the height and width of the input data, respectively. *P* is the padding size, and *S* is the stride size. *KH* and *KW* represent the width and height of the filter, respectively.

The activation functions applied to the output values of the convolutional and pooling layers include sigmoid, tanh and ReLU functions. When the CNN is operating, the learning rate may decrease as the gradient value decreases during the learning process. Therefore, CNN mainly uses the ReLU function as an activation function. In addition, a fully connected layer exists in the CNN, which indicates an existing neural network. That is, if a series of computational processes in the convolutional layer is for information and feature extraction from the input image, the computational process for classification proceeds in the fully connected layer.

Finally, to classify the data, a flattening process is performed to make the outputs of the convolutional layer composed of n channels into one-dimensional data, after which a classification is performed.

## 3. Proposed Method

In this paper, we propose deep-learning-based rainwater-removal image conversion with training pair augmentation. The main contributions of this study are as follows:

(i) To multiply the raindrops-pair dataset using a deep learning model based on the existing dataset. The proposed method can prevent overfitting in subsequent raindrops-removal training by multiplying the insufficient dataset and removing droplets more effectively.
(ii) To select a high-quality dataset that is easier to learn by determining the quality of the multiplied raindrops using CNN.
(iii) To remove raindrops by teaching a deep learning model based on the dataset of high-quality multiplied raindrops determined by CNN and the previously acquired dataset.

The Pix2pix structure was used for image-pair-transformation learning for raindrops removal. In addition, U-net [21] was used for the generator. U-Net was designed to acquire local information in the image-processing field. Whereas conventional convolutional networks classify a class for a dataset, U-net classifies a class for a pixel and is an effective model for semantic segmentation [15]. U-net uses a skip connection to gather the local and context information of data together, prevents the loss of low-level information and is excellent in semantic segmentation considering both local and abstract information. It has characteristics that indicate performance [22].

**Figure 4:**
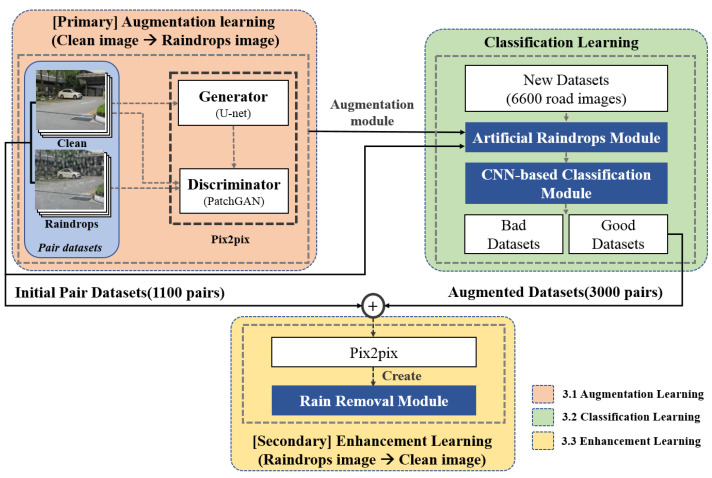
Flowchart of the proposed method.

For the discriminator, 16 × 16 patch GANs were used to determine whether the input image was real or fake in units of patches [23]. The discriminator for each patch had fewer parameters than the discriminator that discriminated the entire image and therefore could output more detailed results because it proceeded with the discrimination for each patch. Figure 4 shows the flowchart of the proposed method. The first learning block refers to primary learning, and virtual water-drop-image generation learning was performed using the initial training image pair. The second learning block represents image classification using a CNN. Finally, the third learning block shows the third supplemented learning process using the augmented learning set, and the classified augmented image and the existing dataset were used for learning.

### 3.1. Augmentation Learning to Obtain Water-Drop-Image Datasets

For deep-learning networks, it is necessary to obtain an appropriate number of training datasets. If the training dataset is insufficient, the training is not performed properly; therefore, the raindrops are not removed properly, and if the training dataset is excessively trained using insufficient training dataset, overfitting occurs. Therefore, it is important to obtain a sufficient amount of training datasets for various environments. However, for Pix2pix, it is difficult to obtain pairs of image sets for sufficient training data. In particular, it is cumbersome to directly photograph a pair of images of the raindrops pattern in various environments.

Qian et al. obtained a dataset using two glasses with the same background to obtain a pair of raindrops images [24]. Glass was applied to the lens to avoid the misalignment of the rays because it has a refractive index different from that of air. In this case, the first image captured through the glass with raindrops and the second image captured through the glass without raindrops were used to obtain paired datasets. Although this method can obtain datasets with high resolution, it is time-consuming because pictures for each different background must be taken. In addition, Takahashi obtained a training dataset using alpha mixing [25]. Alpha blending is a process of creating a new mixed image by combining translucent and background images. It uses the principle that objects appear in the background image by mixing the object distortion image and general background image. However, the after image of the object distortion image used for learning remains in the generated image through the alpha mixing.

Therefore, in this study, to compensate for the lack of training data, the Pix2pix learning model was used to create the virtual raindrops on an image. A raindrops dataset was obtained by forming raindrops on 1100 pairs of existing data images and approximately 6600 general road-view images [26]. The input size of the image is 256 × 256 for the training. For learning, the batch size of Pix2pix was set to 3, and the number of repetitions was set to 400. This dataset was inputted into the Pix2pix model to form the virtual raindrops. However, the activation function of the discriminator was set to Leaky ReLU, not a sigmoid function, to prevent gradient loss in the optimization of the loss function during training [27]. In addition, the gradient of the loss function was continuously updated during the learning process, which could result in different batch-unit data distributions for each layer. Therefore, the data distribution was adjusted by adding batch normalization, and overfitting prevention during the learning process was pursued [28].

**Figure 5:**
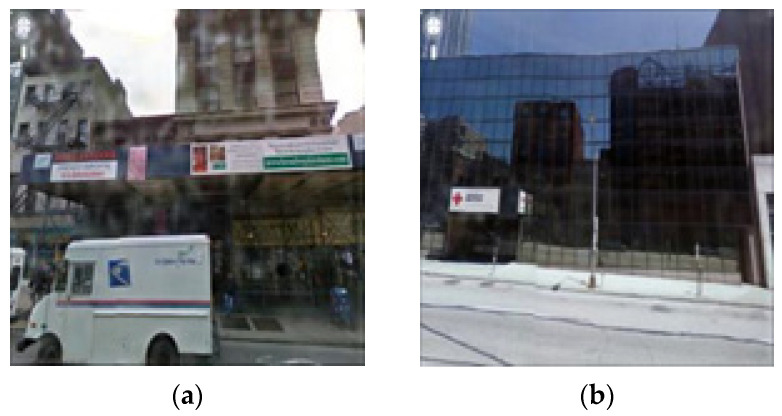
Results of the first raindrops generation training: (**a**) image with pseudo-raindrops and (**b**) image without raindrops.

However, as mentioned earlier, owing to the insufficient dataset, the droplet generation results were different. As shown in Figure 5, the generated raindrops were relatively clear in certain images, whereas the raindrops generation effect was insignificant in specific images.

### 3.2. Classification Learning of Virtual Datasets Using the CNN Model

**Figure 6:**
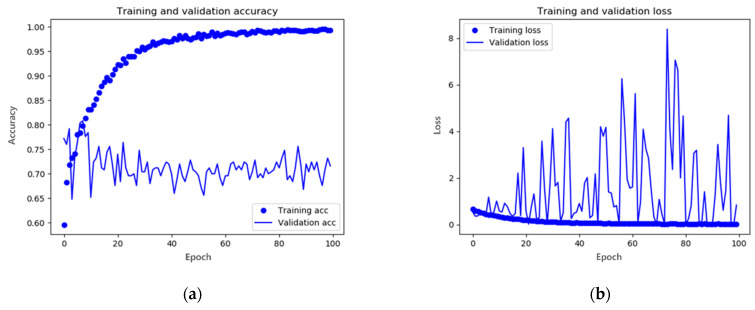
Results of the initial CNN model training: (**a**) accuracy and (**b**) loss. In *x*-axis, epoch represents the number of repetitions for training.

To improve the learning performance, it is necessary to utilize images in which the raindrops are relatively intact. However, it is exhausting to visually identify all the generated images. Therefore, we used a CNN model, which is a deep learning network useful for image discrimination, to classify good raindrops-formation images. To learn the CNN model that classified the corresponding image results, 1100 image pairs used in the primary augmentation learning were used, and according to the classification learning, good and non-good images were classified among 6600 raindrops-formation data. The existing 1100 images of raindrops were added to approximately 3000 images of raindrops and used for secondary learning to enhance raindrops images. The CNN model consists of four convolutional layers with the ReLU activation function, and RMSprop was used as the gradient loss optimization method [29]. The epoch 100 was set for training. Consequently, the learning figures shown in Figure 6 were obtained.

Figure 6a shows the accuracy based on the number of training times; the training accuracy was the accuracy of the training data set, and the validation accuracy was the accuracy of the intermediate test dataset. Both values should be close to one for good performance. However, in the figure, it can be seen that the accuracy of the training dataset was high, whereas the accuracy of the test dataset was slightly different from the learning accuracy. It can be seen that overfitting occurred due to the lack of the training data set. Figure 6b shows the degree of image loss. Training loss means training loss; validation loss means intermediate test loss, and the closer it is to zero, the better the performance. However, in Figure 6b, as in Figure 6a, it can be seen that the degree of learning loss converges to 0, but the degree of test loss fluctuates sharply. To improve this, the data images were augmented by applying left and right, vertical inversion and constant angular rotation to the images classified into the comparison group, and then learning was carried out. Consequently, the numerical values shown in Figure 6 were obtained.

**Figure 7:**
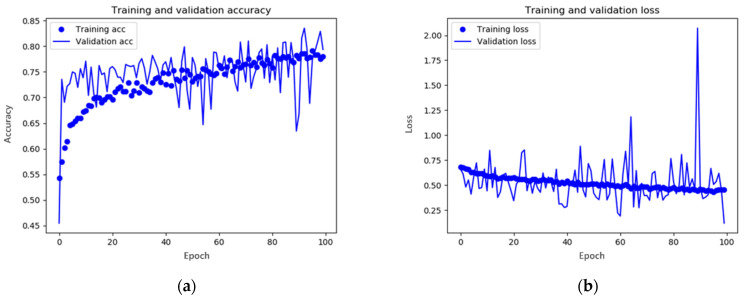
Results of the data-augmented CNN model training: (**a**) accuracy and (**b**) loss. In *x*-axis, epoch represents the number of repetitions for training.

Similar to Figure 6, Figure 7a,b show the corrected accuracy and loss, respectively. Although the performance of the learning accuracy and learning loss decreased slightly compared to the initial model values, it can be seen that the accuracy and loss of the test image converged to the learning numerical value. For the test accuracy, it can be seen that an accuracy of approximately 80% increased by 10%, and the test loss also had a relatively stable fluctuation compared to the previous one. Therefore, raindrops-generation classification was performed using a CNN model with primary data augmentation.

### 3.3. Enhancement Learning to Form a Rain Removal Model

The classified images by CNN are added to the input with the existing dataset as learning images in the Pix2pix to supplement the learning to remove raindrops. Augmented data obtained through primary augmentation learning and CNN-based classification learning were used to train the final droplet-removal model. As shown in Figure 3, the good virtual raindrops-image set separated by the CNN classification model from the existing obtained image set was used for the primary augmentation learning, and the resulting image of the augmentation learning module was provided as input to the Pix2pix model used for the final droplet-removal learning.

In the final learning step, the goal was to remove raindrops; therefore, unlike the primary learning, the order of the image pairs must be changed and provided as input. The learning environment was learned by specifying a batch size of 3, as in the primary learning, and the activation function was a hyperbolic tangent. In addition, the final epoch was set to 100, and the training was performed.

## 4. Simulation Results

To evaluate the performance of the proposed model, the conventional two models were used. The models used for comparison were the region-weighted method (RWM) [30] and Pix2pix. In addition, comparison between models may depend on the learning epoch. Therefore, in the evaluation, both the existing and learning models were compared under the same conditions by setting the learning epoch to 100.

### 4.1. Subjective Assesment

**Figure 8:**
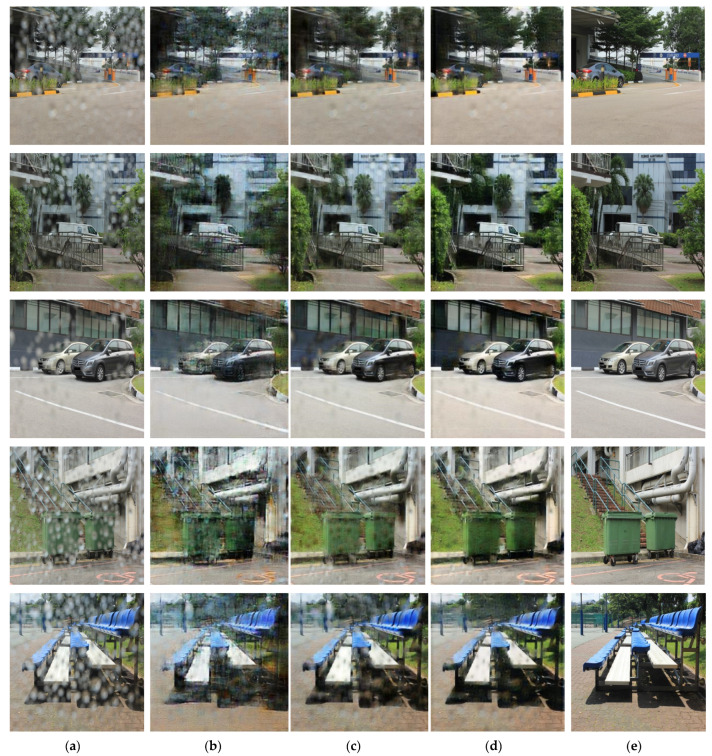
Raindrops images and resulting images for bright outdoor scene: (**a**) original water-drop images, (**b**) images using the region-weighted method, (**c**) images using the Pix2pix method, (**d**) images using the proposed method and (**e**) target images.

**Figure 9:**
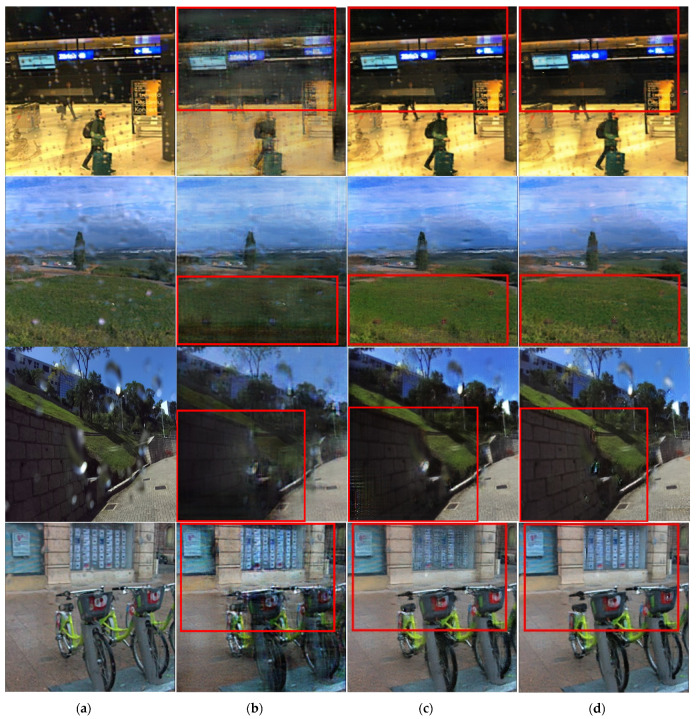
Raindrops images and resulting images for dim outdoor scene: (**a**) original water drop image, (**b**) results using the region-weighted method, (**c**) results using the Pix2pix method and (**d**) results using the proposed method.

**Figure 10:**
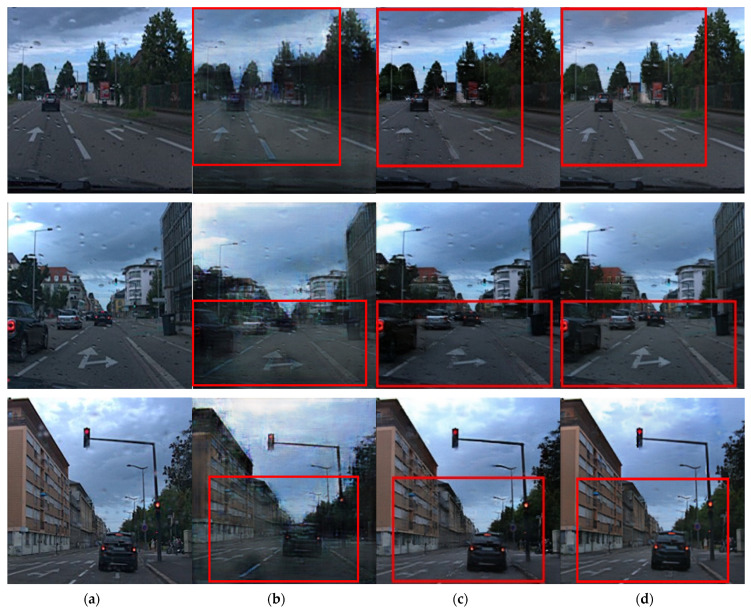
Raindrops images and resulting images for road view: (**a**) original water drop image, (**b**) results using the region-weighted method, (**c**) results using the Pix2pix method and (**d**) results using the proposed method.

Figure 8, Figure 9 and Figure 10 show the results of removing raindrops from a pair of raindrops images using RWM, the existing Pix2pix method and the proposed method. The test dataset consisted of outdoor and road images in bright and dim environments. Because the results of the droplet removal may differ depending on the difference in each environment, the results were compared for each environment.

Figure 8 shows the results of the water-drop-removal treatment for an outdoor image that was not used for learning, and a large amount of rainwater was found. Based on the comparison images shown in Figure 8b–d, it was confirmed that raindrops on roads, trees, vehicles and buildings were erased relatively more clearly in Figure 8d than in Figure 8b,c using the conventional method. There was a substantial difference in the water-drop-removal performance compared to that of the existing method for chairs and trash cans. In particular, the detection performance for objects can be expected to improve by sharpening the boundary between the object and the surrounding background.

Figure 9 and Figure 10 compare the results of droplet removal using another dataset [31]. For the test dataset, there were no ground-truth images. Because the corresponding dataset consisted of raindrops images acquired in various environments, it was possible to compare them in terms of water-drop-removal performance and sharpness improvement, depending on the environment.

Figure 9 shows the difference in the performance of the relative droplet-removal and boundary-area expression compared to the existing Pix2pix method and the region-weighted cycleGAN method. The raindrops removal was improved in all images shown in Figure 9d compared with those in Figure 9b,c. In Figure 9b,c, the red boxes, the glass field of the second figure, the wall of the third figure and the bicycle and background patterns of the fourth figure exhibited large differences in texture and boundary features compared with those in the input images. In contrast, in the results shown in Figure 9d, the original boundary features were clearly shown in the area where the raindrops of the input images were removed. It can be observed that the relative sharpness increased in the areas marked with red boxes.

Figure 10 shows the results of the models for application to road-driving images in a low-brightness environment. Each result shows the result of the conversion in an image wherein the droplet pattern was not severe. Figure 10 shows an image with a small amount of rainwater, and the rainwater-removal effect was insignificant. However, the existing technique partially deleted or distorted important road lane information.

When comparing the results in the red box in Figure 10b–d, the degree of improvement in raindrops removal is at a similar level. However, it was confirmed that information distortion was alleviated for the lane part of the image shown in Figure 10d, and the details of the lanes and arrows were more distinct than those in Figure 10b,c. In addition, the details of the surrounding buildings were expressed more clearly.

In the case of Figure 10, because the results of the outdoor driving images are shown, when the proposed model is applied to an autonomous vehicle, it is easy to identify the driving line on the road. Overall, the proposed model not only has better water-drop-removal performance than the existing model but also improves the details of lanes, buildings and objects and improves distortion. Thus, when mounted on a car, driving ability in bad weather conditions can be improved, and it shows that the object detection ability can also be improved.

### 4.2. Objective Assessment

To compare the quality of removed raindrop images, we used 10 total metrics that consisted of mean squared error (*MSE*), root mean squared error (*RMSE*), Erreur Relative Globale Adimensionnelle de Synthèse (*ERGAS*) [32], relative average spectral error (*RASE*) [33], spectral angle mapper (*SAM*) [34], peak signal-to-noise ratio (*PSNR*) [35], universal quality image index (*UQI*) [36], spatial correlation coefficient (*SCC*) [37], block sensitive-peak signal-to-noise ratio (*PSNR-B*) [38]. The evaluation metrics mentioned above measure quality by comparing the reference image with the resulting image of each method. The lower score of *MSE*, *RMSE*, *ERGAS*, *RASE* and *SAM* indicates the better image quality. On the other hand, the higher score of *PSNR*, *PSNR-B*, *UQI* and *SCC* indicates the better image quality.

*MSE* is called the mean squared error, and it is a metric that is calculated as the average value after squaring the difference between the actual value and the error. *MSE* is widely used as an evaluation method for regression models, but it is characterized by being sensitive to outliers because the loss increases in proportion to the square as the error increases. Its mathematical definition is:

(7) $$MSE=\frac{1}{n}\overset{n}{\underset{i=1}{\sum }}{\left(x_{i}-y_{i}\right)}^{2}\;$$

where *x* and *y* are the original image and test image, respectively. *n* is the number of pixels.

*RMSE* is used to solve this problem. The *RMSE* is a value obtained by taking the root of the *MSE*, correcting the error rate of the simple sum of squared errors, and is widely used as a representative regression measure. Its mathematical definition is:

(8) $$RMSE=\sqrt{MSE}$$

*ERGAS* is an index that quantifies the difference in absolute values of the pixel values of the original multispectral image and the fusion image. Its mathematical definition is:

(9) $$ERGAS=100\frac{h}{l}\sqrt{\frac{1}{N}\overset{N}{\underset{i=1}{\sum }}\left(\frac{RMSE^{2}\left(B_{i}\right)}{M_{i}^{2}}\right)}$$

where *h* and *l* are the resolution of the high- and low-spatial-resolution images, respectively. *M_i_* is the mean radiance of each spectral band involved in the fusion. *B_i_* is the spectral bands of the original multispectral image.

*RASE* is an evaluation index showing spectrum average performance. Its mathematical definition is:

(10) $$RASE=\frac{1}{M}\sqrt{\frac{1}{N}\sum_{i=1}^{N}RMSE^{2}\left(B_{i}\right)},$$

where *M* is the mean radiance of the *N* spectral bands (*B_i_*) of the original multispectral image.

*SAM* is an index that quantifies the distance difference between the pixel values of the original multispectral image and the fusion image after expressing them as vectors. Its mathematical definition is:

(11) $$SAM=\cos^{-1}\frac{\sum_{i=1}^{nb}t_{i}r_{i}}{\sqrt{\sum_{i=1}^{nb}t_{i}^{2}}\sqrt{\sum_{i=1}^{nb}r_{i}^{2}}}$$

where *nb* is the number of bands in the image, and *t* and *r* are the reference spectra and the spectra found at each pixel, respectively.

*PSNR* represents the power of noise relative to the maximum power that a signal can have. It is mainly used to evaluate quality loss information in video or video compression. *PSNR* can be calculated using the mean squared error (*MSE*) without considering the power of the signal. Its mathematical definition is:

(12) $$PSNR=10\log_{10}\left(\frac{255^{2}}{\sqrt{MSE}}\right)$$

*PSNR-B* produces objective judgments that accord with observations. The *PSNR-B* modifies *PSNR* by including a blocking effect factor. The equation of *PSNR-B* is as follows.

(13) $$BEF\left(y\right)=\eta \cdot \left[D_{B}\left(y\right)-D_{B}^{c}\left(y\right)\right]$$

(14) $$\eta =\{\begin{array}{cc} \frac{\log_{2}B}{\log_{2}\left(\min\left(N_{H},N_{v}\right)\right)}, & if\;D_{B}\left(y\right)>D_{B}^{c}\left(y\right)\\ 0, & otherwise \end{array}$$

where BEF is the blocking effect factor of test image *y.* $N_{H}$ and $N_{v}$ are the horizontal and vertical dimensions of the $N_{H}\times N_{v}$ image.

(15) $$MSE\text{-}B\;=MSE+BEF_{Tot}\left(y\right)$$

(16) $$BEF_{Tot}\left(y\right)=\overset{K}{\underset{k=1}{\sum }}BEF_{k}\left(y\right)$$

(17) $$PSNR\text{-}B=10\log_{10}\frac{255^{2}}{MSE\text{-}B}$$

where *MSE-B* is the means square error with the blocking effect factor for the original image and test image. $BEF_{Tot}$ is the total blocking effect factor.

*UQI* is an index that quantifies image correlation, bias and pixel difference using the regional mean and deviation of pixel values of the original multispectral image and the fusion image. Its mathematical definition is:

(18) $$UQI=\frac{\sigma_{xy}}{\sigma_{x}\sigma_{y}}\cdot \frac{2\bar{x}\bar{y}}{{\bar{x}}^{2}+{\bar{y}}^{2}}\cdot \frac{2\sigma_{x}\sigma_{y}}{\sigma_{x}^{2}+\sigma_{y}^{2}}$$

where $\sigma_{x}$ is the standard deviation of original image *x*, and $\sigma_{x}$ is the standard deviation of test image *y*. $\sigma_{xy}$ is the covariance of *x* and *y*. $\bar{x}$ and $\bar{y}$ are the mean of *x* and *y*, respectively.

*SCC* reflects the indirect correlation based on the spatial contiguity between any two entities. Its mathematical definition is:

(19) $$SCC=\frac{\sum_{i=1}^{n}\left(X\left(s_{i}\right)-\bar{X}\right)\left(Y\left(s_{i}\right)-\bar{Y}\right)}{\sqrt{\sum_{i=1}^{n}{\left(X\left(s_{i}\right)-\bar{X}\right)}^{2}}\sqrt{\sum_{i=1}^{n}{\left(Y\left(s_{i}\right)-\bar{Y}\right)}^{2}}}$$

where *X* and *Y* are the high-frequency components of the original image *x* and the test image *y*. $\bar{X}=\frac{1}{\left|s\right|}\overset{\left|s\right|}{\underset{i=1}{\sum }}X\left(s_{i}\right)$, |s| is the cardinality of *S* and similarly for $\bar{Y}$.

**Figure 11:**
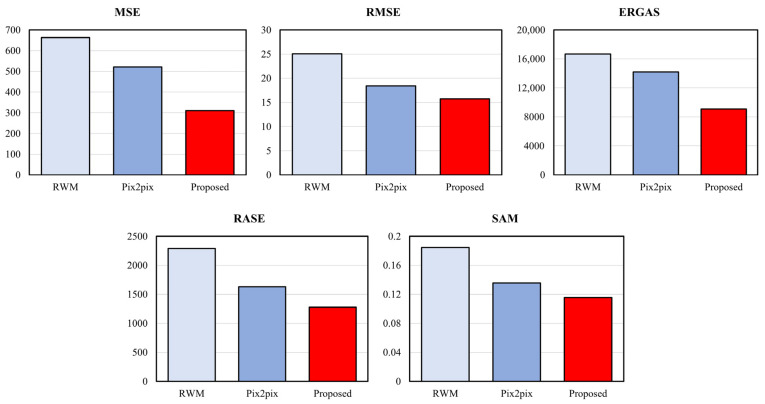
Comparison of full reference image quality assessment metrics (lower score is better).

**Figure 12:**
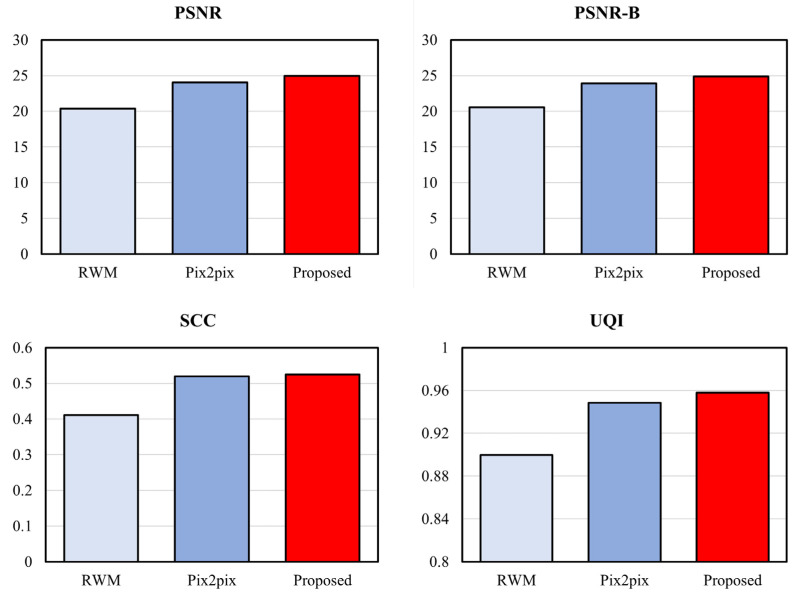
Comparison of full reference image quality assessment metrics (higher score is better).

Table 1 and Table 2 and Figure 11 and Figure 12 show the scores of the full reference image quality assessment between the original image and the resulting image. The results show that the image quality of the proposed method is superior to that of other methods.

## 5. Conclusions

In this study, we proposed an image conversion processing method to effectively remove raindrops from camera lenses used in autonomous vehicles. The proposed method was solved by learning the first-generation model based on an existing dataset to complement the insufficient dataset based on the Pix2pix deep learning model and then proceeding with additional learning with the existing dataset using the raindrops formed through this. In addition, it was confirmed that the virtual data generated by adding CNN was effectively classified. It was confirmed that the raindrops-removal performance was better than that of the model using the existing dataset using the proposed method. This is considered to be a more effective method for raindrops removal than existing methods.

## Figures and Tables

**Table 1:** Comparison of full reference image quality assessment metrics (lower score is better).

	MSE	RMSE	ERGAS	RASE	SAM
Original	0	0	0	0	0
RWM	662.730	25.043	16,681.040	2287.944	0.184
Pix2pix	520.644	18.433	14,219.890	1628.504	0.136
Proposed	310.662	15.747	9096.109	1276.635	0.116

**Table 2:** Comparison of full reference image quality assessment metrics (higher score is better).

	PSNR	PSNR-B	UQI	SCC
Original	Inf	Inf	1	1
RWM	20.380	20.564	0.900	0.411
Pix2pix	24.030	23.922	0.949	0.520
Proposed	24.980	24.887	0.958	0.524

## Data Availability

Not applicable.
